# Hospitalizations and Deaths Associated with Diarrhea and Respiratory Diseases among Children Aged 0–5 Years in a Referral Hospital of Mauritania

**DOI:** 10.3390/tropicalmed3030103

**Published:** 2018-09-17

**Authors:** Mohamed Lemine Cheikh Brahim Ahmed, Abdellahi Weddih, Mohammed Benhafid, Mohamed Abdellahi Bollahi, Mariem Sidatt, Khattry Makhalla, Ali H. Mokdad, Jorg Heukelbach, Abdelkarim Filali-Maltouf

**Affiliations:** 1 Department of Biology, University Mohammed V, Rabat 10010, Morocco; filalimaltouf@gmail.com; 2 Department of Virology, National Institute of Hygiene, Rabat 10010, Morocco; benhafidm@yahoo.fr; 3 Institut National de Recherche en Santé Publique (INRSP), Nouakchott 2373, Mauritania; boullahi01@gmail.com; 4 Ministry of Health and University of Nouakchott, Department of Pediatrics, Nouakchott 2373, Mauritania; aoueddih@gmail.com (A.W.); mariemsi@hotmail.fr (M.S.); drkhattry2006@yahoo.fr (K.M.); 5 Institute for Health Metrics and Evaluation, University of Washington, Seattle, WD 98195, USA; mokdaa@uw.edu; 6 Department of Community Health, School of Medicine, Federal University of Ceará, Fortaleza CE60430-140, Brazil; heukelbach@web.de; 7 College of Public Health, Medical and Veterinary Sciences, Division of Tropical Health and Medicine, James Cook University, Townville, QLD 4810, Australia

**Keywords:** respiratory diseases, diarrhea, hospitalizations, children, Mauritania, deaths

## Abstract

Diarrhea and respiratory diseases are the leading causes of morbidity and mortality among <5-year-olds worldwide, but systematic data are not available from Mauritania. We conducted a hospital-based retrospective study. Data on admissions to Mauritania’s National Referral Hospital (the main pediatric referral center in the country), due to diarrhea and respiratory diseases, during 2011–2014, were analyzed. A total of 3695 children <5 years were hospitalized during this period; 665 (18.0%) due to respiratory diseases, and 829 (22.4%) due to diarrhea. Case fatality rates in the respiratory diseases and diarrhea groups were 18.0% (120/665) and 14.1% (117/829), respectively. The highest frequency of deaths due to diarrhea occurred in the age group 2–5 years (16/76; 21.0%), and due to respiratory diseases in the age group 6–12 months (32/141; 22.6%). We conclude that case fatality rates caused by respiratory diseases and diarrhea are extremely high in children hospitalized at the National Referral Hospital. These data call for intensified efforts to reduce deaths among hospitalized Mauritanian children, and also for integrated control measures to prevent and reduce the burden of both diseases. Additional studies are needed to show the effectiveness of the introduction of vaccination programs for pneumococcal diseases and rotavirus infection in the child population, which were launched in November 2013 and December 2014, respectively.

## 1. Introduction

Diarrhea and respiratory diseases are the leading causes of morbidity and mortality among children under five years of age, over the world [1,2]. Despite the decline in the burden of diarrhea and respiratory diseases since 2005, they still pose a major public health burden [3]. Both of these infectious diseases have always been in the 10 top causes of deaths among children, especially in low- and middle-income countries [4]. Globally, rotavirus and *Streptococcus pneumoniae* are the most common causes of severe diarrhea and respiratory infections in children, contributing to 28% and 18% of diarrhea cases and respiratory infections, respectively [2,5]. Given the high morbidity and mortality of diseases caused by these pathogens, the World Health Organization (WHO) recommended vaccination with rotavirus and pneumococcal conjugate vaccines (PCV13) [6,7].

Mauritania is an Arabic–African country, located between North and West Africa, on the Atlantic Ocean. It has a population of approximately 3.5 million and more than one-third of the population lives in Nouakchott territory [8]. The number of children aged 0–5 years was estimated to be 17% of the general population in 2015 [9]. In the Arabic region, including Mauritania, respiratory diseases and diarrhea account for about 50% of all post-neonatal deaths [10].

The Global Burden of Diseases study estimated that, in Mauritania, mortality from diarrhea was 10.5% (95% CI: 7.6%–14.4%) and that from respiratory disease was 12.8% (9.4%–17.1%) of the total deaths, among children <5 years [3]. From 2005 to 2016, the mortality associated with these diseases in the country decreased by 33.5% and 12.2%, respectively. In spite of this decrease, respiratory diseases are still the second leading cause of deaths [3]. Diarrhea also remains the third leading cause of deaths among children aged 0–5 years. The death rate per 100,000 was estimated at 110.3 in 1990, 85 in 2005 and 50.2 in 2016 [3].

There are no systematic baseline data available on the burden of diarrhea and respiratory diseases among children aged 0–5 years, in Mauritania. The aim of this study was to identify the proportion and case fatality rates of diarrhea and respiratory diseases in children of age 0–5 years, who have been hospitalized during 2011–2014, at the largest pediatric referral hospital in Mauritania.

## 2. Materials and Methods

### 2.1. Setting

The study was performed in Nouakchott territory, which represents 1/3 of Mauritania population, at the National Referral Hospital—the main pediatric referral hospital in Mauritania. The hospital was constructed in 1966 with more than 450 beds. It was the first and largest tertiary hospital in Mauritania and aimed to receive severely-ill patients, transferred from any health facilities throughout the country. The pediatric services at this hospital have a capacity of about 100 beds with 1000–1500 hospitalizations annually, and receive about 300–500 high-risk children, transferred annually from outside of Nouakchott, to be admitted to this hospital.

### 2.2. Inclusion Criteria and Variables

We conducted a retrospective study based on analyses of the registries of pediatric admissions to the hospital. All hospitalizations of children aged 0–5 years due to respiratory diseases or diarrhea, from 1 January 2011 to 31 December 2014, were included. We aimed to describe the status before effective implementation of the vaccination programs, whose significant effects were expected to start after this period. Case definitions are presented in Table 1.

Data available included age, sex, admission and discharge dates, diagnosis on admission and discharge, and outcome (deceased, discharged). If the patient had a diagnosis of both diarrhea and respiratory diseases, we recorded it as both cases, but hospitalization was counted only once.

### 2.3. Data Analysis

Data were entered into Microsoft Office Excel 2007 spreadsheets, checked for entry-related errors and analyzed using Statistical Package for the Social Sciences (SPSS, version 22) (IBM, SPSS Inc, Chicago, IL, USA). Death and discharge rates were calculated based on the total number of hospitalizations in the corresponding diagnostic groups. Statistical significance of differences between groups was evaluated by the chi-squared test.

## 3. Results

### 3.1. General Characteristics 

From 1 January 2011 to 31 December 2014, a total of 3695 children aged 0–5 years was hospitalized. Children aged 0–5 months accounted for 38%, those aged 0–11 months for 50.4%, and those aged 0–23 months for 73.4% of all hospitalized children. Most children were living in Nouakchott territory (81.9%), while those transferred from other regions accounted for 18.1%. Vaccination cards were complete and up-to-date in 55% of cases, incomplete in 35% of cases, and absent or not clear in 10%.

From these hospitalizations, 665 (18.0%) were admitted due to respiratory diseases, and 829 (22.4%) due to diarrhea (Table 2). The highest relative frequency of hospitalizations that occurred in <6-month-olds were 47.0% for respiratory diseases, and 31.9% for diarrhea. A total of 68% of all hospitalizations were treated with an antibiotic. Infectious gastroenteritis accounted for 65% of diarrheal diseases, and 78% of respiratory diseases were related to bronchopneumonia.

**Figure 1:**
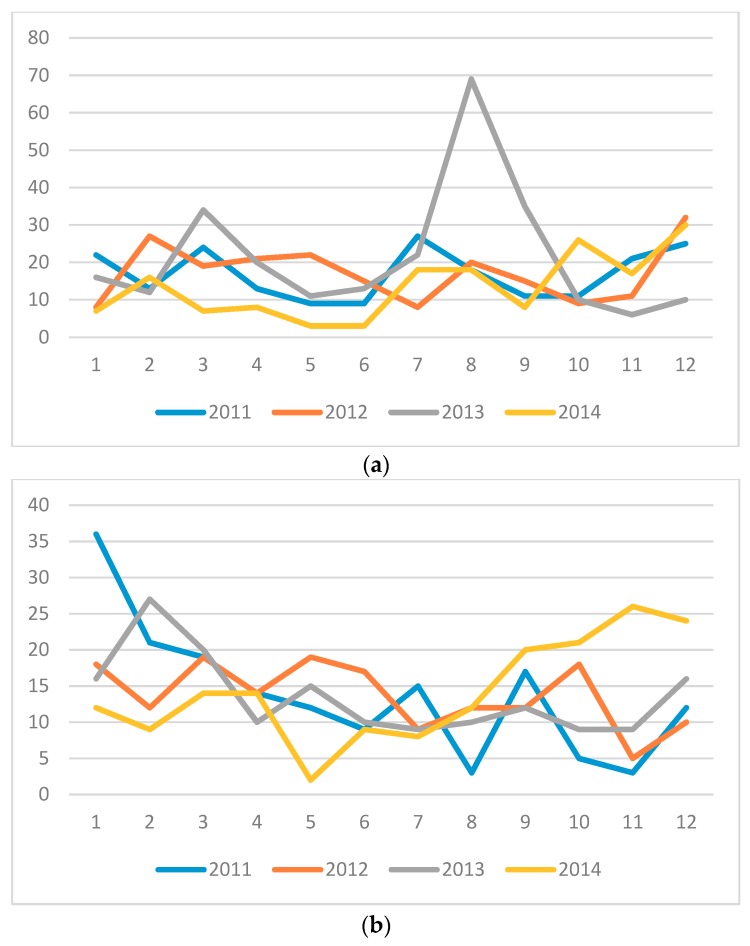
(**a**) Monthly variation of cases of diarrhea, stratified by year. X-axis: months of the year; y-axis: absolute number of cases. (**b**) Monthly variation of cases of respiratory diseases, stratified by year. X-axis: months of the year; y-axis: absolute number of cases.

There was no clear seasonal pattern of diarrhea, with the exception of a prominent peak in August 2013 (*n* = 69; Figure 1a). The frequency of cases of respiratory diseases varied, throughout the years and months, without any clear seasonal patterns (Figure 1b). The highest number of respiratory cases was observed in January 2011 (*n* = 36).

### 3.2. Outcomes and Associated Factors

About 1/7 of the hospitalized children with diarrhea or respiratory diseases died (Table 3). Case fatality rates were similarly high for both groups (Table 3). The highest frequency of deaths after admission due to diarrhea occurred in the <6-month-olds (15.9%) and in the >2-year-olds (21.0%, Table 4). The case fatality rates due to respiratory disease were highest in infants, with 19.5% in <6-month-olds, and 22.6% in 6–11-month-olds. (Table 4).

Table 4 presents case fatality rates due to both diarrhea and respiratory diseases, stratified by age, sex and year of submission. Case fatality rates were slightly, but not significantly, higher in females, in the diarrhea group, and slightly higher in males, in the respiratory disease group. There was a considerable decrease in case fatality rates in both groups in the year 2014, as compared to the previous years.

## 4. Discussion

To the best of our knowledge, this is the first systematic, hospital-based study on the burden of diarrhea and respiratory diseases among hospitalized children in Mauritania. Our study was conducted in the highest populated city in Mauritania. The data show that both diseases were important causes for hospitalization, and that they caused extremely high case-fatality rates, in the child population. These results are in line with previous studies that have shown that diarrhea and respiratory diseases contribute to a high morbidity and mortality among pediatric patients in developing countries [2,11,12,13]. 

At the time of this study, there was only one referral hospital in the country, particularly in Nouakchott city, with poor quality of care services, in addition to poor general hygiene conditions in the city. Being a referral center, the hospital receives the most complicated cases from all different regions of the country, annually. These factors may explain the extremely high case fatality rates in pediatric patients hospitalized with diarrhea and respiratory diseases. In fact, according to a study conducted in 2012, 2150 people, including 1700 children less than five years of age, die each year from diarrhea in Mauritania, and 90% of these deaths are directly attributable to the poor quality of water, sanitation, and hygiene [14]. In addition, many patients present to the health system after a relatively high time lapse since the start of symptoms, as a result of difficult access to the health system, and also a low awareness of the urgency of these conditions, in the child population.

Despite the progress made by the government in order to reduce the morbidity and case fatality of both diarrhea and respiratory diseases, such as the promotion of effective interventions and the improvement of available treatment and vaccines, the Ministry of Health of Mauritania still lists respiratory diseases and diarrhea as the first and second leading causes of death, respectively, among children aged 0–5 years [15]. The Ministry of Health of Mauritania officially introduced pneumococcal vaccine (PCV13) in November 2013 and rotavirus vaccine (Rotarix) in December 2014. As our study mostly covers the period before the effective introduction of these vaccine programs, and some time is needed until the effect can be observed on a population level, additional studies are needed to show the effect of these interventions on child health in the country. In addition, two new hospitals were inaugurated in Nouakchott, in 2013, with a possible positive effect on health outcomes.

Of the 55% of cases in which a vaccination card was available, 60% were vaccinated with PCV13 by December 2014. The decrease in the number of hospitalizations due to respiratory diseases in 2014, as compared to the previous years (as was observed in our study), might already indicate the first effect of PCV13 vaccine introduction in 2013 in Mauritania. However, it could also be expected that the most significant effects of vaccination programs will only be observed after the study period. In fact, in other countries, a significant reduction of morbidity and mortality has been reported after the introduction of vaccine programs. For example, in South Africa, pneumococcal vaccination programs contributed to a reduction of 69% of the incidence of invasive pneumococcal disease [7]. In Mexico, rotavirus vaccine contributed to a 38% reduction in diarrhea associated with hospitalization [16], and a 50% reduction in diarrhea associated with death [17].

The number of hospitalizations due to diarrheal diseases in the <6-month-olds were the highest among all age groups. In contrast, the highest frequency of hospitalization due to respiratory diseases was among children aged 6–11 months. This finding may further indicate that the introduction of rotavirus vaccine (at age of 6 and 10 weeks) and PCV13 vaccine (at age of 6, 10 and 14 weeks) will have a major impact in reducing the burden of diarrhea and respiratory diseases. Our results were supported by other findings that reported the highest number of hospitalizations was among children aged 0–11 months, in other countries [1,2,18,19]. These two age groups were also the most common age groups hospitalized for a long period, with a minimum of two days of stay at the hospital.

The majority of hospitalizations in our study were for females (57%), and diarrhea-associated deaths were also higher among them, similar to a previous study from Bangladesh [20]. On the other hand, respiratory disease-associated deaths were more common in males than females (20% vs. 16%). This finding is supported by previous studies from low, middle and high-income countries that reported the occurrence of respiratory diseases to be more frequent in males than females [21,22,23]. However, the differences found in our study were not statistically significant, and it is difficult to speculate on reasons for these sex differences based on the nature of the study design.

We did not observe any clear seasonal patterns for either of the diseases, as climatic conditions are similar throughout the year, with the exception of rainfall that almost exclusively occurs in July, August, and September. Singular peaks during different months throughout the years as observed in our study indicate outbreaks, such as a diarrhea outbreak during the rainy season in 2013, especially in Brakna region, with the majority of cases being transferred to the National Hospital in Nouakchott. However, the etiological agent of this outbreak was not known, and the event was only rudimentarily documented in the lay media [24].

Our study was subject to limitations. The analysis of secondary hospital data might present inconsistencies in quantity (such as missing information) and quality (such as diagnostic errors). In addition, the number of variables to be analyzed was limited. Despite these limitations, the analyzed data can be considered as consistent, as they are derived from a single source, and are based on standardized procedures, within the National Referral Hospital, which did not change throughout the study period. As the hospital was the unique referral hospital nationwide, during the study period, data may be considered representative for hospitalized children in the country. However, as this was not a population-based study, we were unable to calculate any hospitalization or incidence rates. We also could not directly conclude on the epidemiological situation of the diseases, under study, in the country. This is because access to the health system is not equal and the rural areas are especially underserved. Therefore, children may have presented at a late stage of the disease with a considerably increased risk of death, and others may have died on their long way to the hospital. As laboratory testing for diarrhea and pneumonia is not provided on a routine basis in Mauritania, and as the study was performed without any funding, information on pathogens was not available.

Difficult access to health facilities and to effective treatment remains a major hindrance to prevention and treatment of childhood diseases. The quality of healthcare services still suffers from some drawbacks, and many children are treated outside health facilities. Primary healthcare facilities are insufficient. Recent studies conducted in Nouakchott reported that less than 44% of children were taken to a healthcare facility, and only 26% of households had access to safe drinking water sources, while 70% of the population had access to improved latrines [9,14]. The situation in rural areas is even worse.

## 5. Conclusions

Our study demonstrated that diarrhea and respiratory diseases accounted for a high number of hospitalizations and deaths among children aged 0–5 years in Mauritania’s main referral hospital. These findings call for intensified efforts, and specific programs and policies, to improve vaccination coverage, access to resources of safe water, intensify health education, and improve access to and quality of healthcare, in order to control and reduce the morbidity and mortality caused by respiratory diseases and diarrhea in children. Clinicians should be alert to early diagnosis and to prevent complications and death. Future studies are needed to show the effectiveness of the introduction of vaccination programs for pneumococcal diseases and rotavirus infection in the child population, since 2013.

## Figures and Tables

**Table 1:** Case definitions of diarrhea and respiratory diseases used for data abstraction (ICD-10 codes).

Diarrhea	Respiratory Diseases
Non-infectious gastroenteritis (K52)	Pneumonia, bronchopneumonia, pneumonitis, acute respiratory infection (J18; J13; J15; J22)
Infectious gastroenteritis (A09)	Bronchitis, acute respiratory distress syndrome, asthma (J06; J20; J22; J80)

**Table 2:** Characteristics of children admitted due to diarrhea and/or respiratory diseases (*n* = 1494).

Characteristics	Diarrhea *n* (%)	Respiratory Diseases *n* (%)	Total *n* (%)
Sex:			
Male	347 (41.8)	296 (44.5)	643 (43)
Female	482 (58.2)	369 (55.5)	851(57)
Age:			
<6 months	264 (31.8)	312 (46.9)	576 (38.6)
6 months <1 year	258 (31.2)	141 (21.2)	399 (26.7)
1 year <2 years	231 (27.8)	109 (16.4)	340 (22.7)
2 years <5 years	76 (9.2)	103 (15.5)	179 (12)
Year of admission:			
2011	203 (24.7)	166 (24.9)	369 (24.7)
2012	207 (24.9)	165 (24.8)	372 (24.9)
2013	258 (28.2)	163 (24.5)	421 (28.2)
2014	161 (22.2)	171 (25.7)	332 (22.2)
Total	829 (55.4)	665 (44.6)	1494 (100)

**Table 3:** Outcome of diarrhea and respiratory diseases and the average of hospitalization periods in children <5 years of age.

Outcome	Diarrhea *n* (%)	Respiratory Diseases *n* (%)
Discharge	712 (85.9)	545 (81.9)
Death	117 (14.1)	120 (18.1)
Average length of stay in days (mean and SD)	5.28 (4.36)	5.66 (4.86)

**Table 4:** Case fatality rates due to diarrhea and respiratory diseases, stratified by sex, age and year of submission.

Variable	Diarrhea Deaths *n* (%)	*P* Value	Respiratory Diseases Deaths *n* (%)	*P* Value
Sex:				
Male	44/347 (12.6)		60/296 (20.2)	
Female	73/482 (15.1)	0.35	60/369 (16.2)	0.16
Age:				
<6 months	42/264 (15.9)		61/312 (19.5)	
6 months <1 year	30/258 (11.6)		32/141 (22.6)	
1 year <2 years	29/231 (12.5)		15/109 (13.7)	
2 years <5 years	16/76 (21.0)	0.74	12/103 (11.6)	0.049
Year of submission:				
2011	22/203 (10.8)		33/166 (19.8)	
2012	41/207 (19.8)		33/165 (20.0)	
2013	39/258 (15.1)		31/163 (19.0)	
2014	15/161 (9.3)	0.58	23/171 (13.4)	0.15

## References

[1] Walker C.L.F., Rudan I., Liu L., Nair H., Theodoratou E., Bhutta Z.A., O’Brien K.L., Campbell H., Black R.E. (2013). Global burden of childhood pneumonia and diarrhoea. Lancet.

[2] Vinekar K, Schaad N., Ber-Lucien M.A., Leshem E., Oboho I.K., Joseph G., Juin S., Dawood F.S., Parashar U., Katz M.A. (2015). Hospitalizations and deaths because of respiratory and diarrheal diseases among Haitian children under five years of age, 2011–2013. Pediatr. Infect. Dis. J..

[3] Global Burden of Disease Collaborative Network, Study 2016 Results (GBD 2016). http://ghdx.healthdata.org/gbd-results-tool/result/6be59e68f0af0a790a5560a10503a421.

[4] Bagherian H., Farahbaksh M., Rabiei R., Moghaddasi H., Asadi F. (2017). National communicable disease surveillance system: A review on information and organizational structures in developed countries. Acta Inform. Med..

[5] Smok B., Zieniewicz-Cieślik K., Smukalska E., Pawłowska M. (2016). Acute diarrhoea induced by rotavirus in children hospitalised in provincial hospital for infectious diseases in Bydgoszcz in 2014 year. Przegl Epidemiol..

[6] UNICEF/WHO Diarrhea: Why Children Are Still Dying and What Can Be Done. http://www.who.int/maternal_child_adolescent/documents/9789241598415/en/.

[7] Von Gottberg A., De Gouveia L., Tempia S., Quan V., Meiring S., Von Mollendorf C., Madhi S.A., Zell E.R., Verani J.R., O’Brien K.L. (2014). Effects of vaccination on invasive pneumococcal disease in South Africa. New Engl. J. Med..

[8] Africa BBC Mauritania Profile Timeline. https://www.bbc.co.uk/news/world-africa-13882166.

[9] UNICEF Enquête par Grappes à Indicateurs Multiples (MICS). https://www.unicef.org/french/statistics/index_24302.html.

[10] Akseer N., Kamali M., Husain S., Mirza M., Bakhache N., Bhutta Z.A. (2015). Strategies to avert preventable mortality among mothers and children in the Eastern Mediterranean Region: New initiatives, new hope. East. Mediterr. Health J..

[11] Malek M.A., Teleb N., Abu-Elyazeed R., Riddle M.S., Sherif M.E., Steele A.D., Glass R.I., Bresee J.S. (2010). The epidemiology of rotavirus diarrhea in countries in the Eastern Mediterranean Region. J. Infect. Dis..

[12] Al-Badani A., Al-Areqi L., Majily A., AL-Sallami S., AL-Madhagi A., Amood A.K. (2014). Rotavirus diarrhea among children in Taiz, Yemen: Prevalence—risk factors and detection of genotypes. Int. J. Pediatr..

[13] Nech M.A., Sejad M.O.A., Dahdi S.A. (2012). Le cancer du poumon à Nouakchott. Expérience du service de pneumologie. Rev. Mal. Respir..

[14] Sy I., Traoré D., Diène A.N., Koné B., Lô B., Faye O., Utzinger J., Cissé G., Tanner M. (2017). Eau potable, assainissement et risque de maladies diarrhéiques dans la Communauté Urbaine de Nouakchott, Mauritanie. Santé Publique.

[15] WHO OMS Stratégie de Coopération: Un Aperçu: Mauritanie. http://apps.who.int/iris/handle/10665/136942.

[16] Esparza-Aguilar M., Gastañaduy P.A., Sánchez-Uribe E., Desai R., Parashar U.D., Richardson V., Patel M. (2013). Diarrhoea-related hospitalizations in children before and after implementation of monovalent rotavirus vaccination in Mexico. Bull. World Health Organ..

[17] Gastañaduy P.A., Sánchez-Uribe E., Esparza-Aguilar M., Desai R., Parashar U.D., Patel M., Richardson V. (2013). Effect of rotavirus vaccine on diarrhea mortality in different socioeconomic regions of Mexico. Pediatrics.

[18] Andrews J.R., Leung D.T., Ahmed S., Malek M.A., Ahmed D., Begum Y.A., Qadri F., Ahmed T., Faruque A.S.G., Nelson E.J. (2017). Determinants of severe dehydration from diarrheal disease at hospital presentation: Evidence from 22 years of admissions in Bangladesh. PLoS Negl. Trop. Dis..

[19] Khoury H., Ogilvie I., El-Khoury A.C., Duan Y., Goetghebeur M.M. (2011). Burden of rotavirus gastroenteritis in the Middle Eastern and North African pediatric population. BMC Infect. Dis..

[20] Mitra A.K., Rahman M.M., Fuchs G.J. (2000). Risk factors and gender differentials for death among children hospitalized with diarrhoea in Bangladesh. J. Health Popul. Nutr..

[21] Nagayama Y., Tsubaki T., Nakayama S., Sawada K., Taguchi K., Tateno N., Toba T. (2006). Gender analysis in acute bronchiolitis due to respiratory syncytial virus. Pediatr. Allergy Immunol..

[22] Falagas M.E., Mourtzoukou E.G., Vardakas K.Z. (2007). Sex differences in the incidence and severity of respiratory tract infections. Respir. Med..

[23] Jensen-Fangel S., Mohey R., Johnsen S.P., Andersen P.L., Sørensen H.T., Østergaard L. (2004). Gender differences in hospitalization rates for respiratory tract infections in Danish youth. Scand. J. Infect. Dis..

[24] Mauritanie: Des cas de Diarrhée Accompagnée de Vomissements, Recensés au Brakna. http://cridem.org/C_Info.php?article=647060.

